# The impact of working in immersive theatre on actors’ health

**DOI:** 10.1093/occmed/kqag086

**Published:** 2026-09-04

**Authors:** L Rayner, J Grieshofer

**Affiliations:** Division of Surgery and Interventional Science, University College London, Gower Street, London, WC1E 6BT, UK; Division of Psychology and Language Sciences, University College London, 1-19 Torrington Place, Fitzrovia, London, WC1E 7HB, UK

## Abstract

**Background:**

Actors face significant occupational health challenges, including high rates of physical injury, mental health problems and voice disorders. Immersive theatre is a novel but growing performance genre in which actors must navigate a shared space with the audience. Currently, no research has investigated the effects this working environment has on the health and wellbeing of actors.

**Aims:**

To explore how the unique nature of the immersive theatre working environment may impact the physical, vocal and mental health of actors.

**Methods:**

Qualitative study using semi-structured interviews with immersive theatre actors. Interviews were conducted online, recorded, transcribed and analysed using thematic analysis.

**Results:**

Analysis identified 12 sub-themes grouped into 4 themes: Vocal Health, Physical Health, Mental Health and Audience Management. Key concerns include vocal strain from a high vocal demand in difficult environmental conditions, cognitive overload from constant improvisation in highly stimulating settings, an elevated physical injury risk linked to a hazardous environment and lack of healthcare support, and personal safety concerns whilst working in the same space as an unpredictable audience.

**Conclusions:**

Actors value the creative aspects of immersive theatre but face serious genre-specific occupational health risks. Targeted guidelines are needed for this expanding industry.

## Teaser

Immersive theatre is a novel performance genre in which the boundaries present in traditional theatre settings are removed. This creates unique environmental and occupational challenges for actors, as they must navigate a shared performance space and interact with the audience. A qualitative approach, using thematic analysis of semi-structured interviews, was used to explore the impact of this working environment on the physical, mental and vocal health of actors.

Key learning points **What is already known about this subject:** 

- A high proportion of actors are affected by physical injury, mental health issues and vocal problems.
- Immersive theatre creates distinct environmental and occupational demands unlike those in traditional theatre.
- No prior research has examined the impact of immersive theatre on actor health and wellbeing.

**What this study adds:** 

- The unique nature of immersive theatre has the potential to create a higher risk to the health of actors.
- There are genre-specific risks across four domains: vocal health, mental health, physical health and audience management.
- The actor must also manage challenges and personal safety issues not normally encountered in traditional theatre settings.

**What impact this may have on practice or policy:** 

- Healthcare professionals working with immersive theatre actors should be aware of the specific and cumulative risks in this setting.
- Producers and managers need to ensure that thorough risk assessments are completed and create workplace cultures in which actors can raise health concerns without fear of professional consequences.
- Clear policies on audience management, harassment prevention and incident reporting, consistent with current UK legislation, are required.

## Introduction

Acting is physically, vocally and psychologically demanding work. The prevalence of health-related complaints in a recent study was high, and depression scores in actors were double that of the general population [1]. Work-related pressures, environmental challenges and lack of confidence are cited as causes of mental health issues [2]. Portraying emotionally charged roles requires actors to tap into challenging emotions, which can trigger or reveal personal conflict. In addition, some actor-training techniques may use personal experiences to enact characters, potentially leading to personal distress [3]. Actors face long hours, poor pay, and suffer employment insecurity, which can cause stress and negatively impact mental health [1,4].

In addition to mental health concerns, actors face a high risk of physical injury and vocal health problems. The prevalence of musculoskeletal injury in musical theatre actors is high, with 55% of Broadway performers [5] and 46–65% of West End theatre performers sustaining at least one injury [6,7]. A study of Australian musical theatre performers noted a high prevalence of medical attention injuries ranging from 39.5 to 96.4% depending on the show and region [8]. Actors are considered elite vocal performers and rely on a fully functioning voice as a primary part of their occupation. The prevalence of voice disorders in actors is higher than in the general population, linked to a range of intrinsic and extrinsic risk factors [9].

Immersive theatre is a form of contemporary performance where the traditional theatre setting is disrupted, and the actors and audience interact in a shared reality. There is considerable variation in format, but in essence, the traditional stage boundary or ‘fourth wall’ is removed or blurred, allowing the audience greater freedom to engage with the performance compared to traditional theatre settings [10,11]. Actors must navigate this environment, and despite the rapid growth of this genre, no research has examined the occupational health implications. This study aims to address this gap by exploring how immersive theatre affects actors’ physical, vocal and mental health.

## Methods

A qualitative research methodology using semi-structured interviews was chosen to address the study aims. The study design adhered to the Consolidated Criteria for Reporting Qualitative Research (COREQ) guidelines [12]. Participants were a convenience sample recruited via online adverts and social media targeting UK-based immersive theatre actors. Inclusion criteria were UK residents, aged 18 years or over, with immersive theatre experience within the last 12 months. Recruitment continued until the researchers judged that sufficient data had been generated to support a rich and meaningful analysis [13]. Ten actors participated (four females and six males, with a mean age of 39.1 years). All participants provided informed, written consent.

All interviews were conducted online by the same female researcher (L.R.) using Microsoft Teams. The interviews lasted ∼60 min and were recorded and transcribed verbatim. Transcripts were de-identified with names, locations and companies removed, and participants were assigned anonymous IDs (P01–P10). The interview schedule is provided in Appendix 1 (available as Supplementary material at *Occupational Medicine* Online).

Data were analysed using Braun and Clarke’s six-phase thematic analysis framework: data familiarization; generating initial codes; seeking themes; reviewing themes; defining and labelling themes; and producing the report [14]. The approach was primarily inductive as themes emerged directly from participants’ accounts. However, existing literature on performing arts health informed the interview questions and provided a broad orienting framework, introducing a degree of deductive influence.

L.R.’s background as a senior performing arts physiotherapist provided valuable contextual knowledge but risked interpretive bias towards physical health concerns. To manage this, L.R. maintained reflexive notes documenting key analytical decisions and assumptions, discussed iteratively with J.G. throughout each phase. J.G. holds a PhD examining actors’ mental health and resilience and works clinically with performers. The complementary expertise of the two coders strengthened theme development.

L.R. generated initial codes manually from all transcripts. J.G. independently coded a subset (∼30%) and reviewed all codes and themes. A consensus was reached through discussion. Trustworthiness was supported through dual coding, reflexive documentation and adherence to COREQ.

Ethics approval was granted by University College London (UCL) Ethics Committee (Ethics ID: 22599/002).

## Results

Ten participants were interviewed (Table 1). Four major themes and 12 sub-themes emerged (Figure 1). Participants valued the creative freedom of immersive theatre, as it allows autonomy to shape the performance experience. However, they consistently raised health and safety concerns unique to the genre. Table 2 presents supporting quotations for all themes and sub-themes.

**Figure 1:**
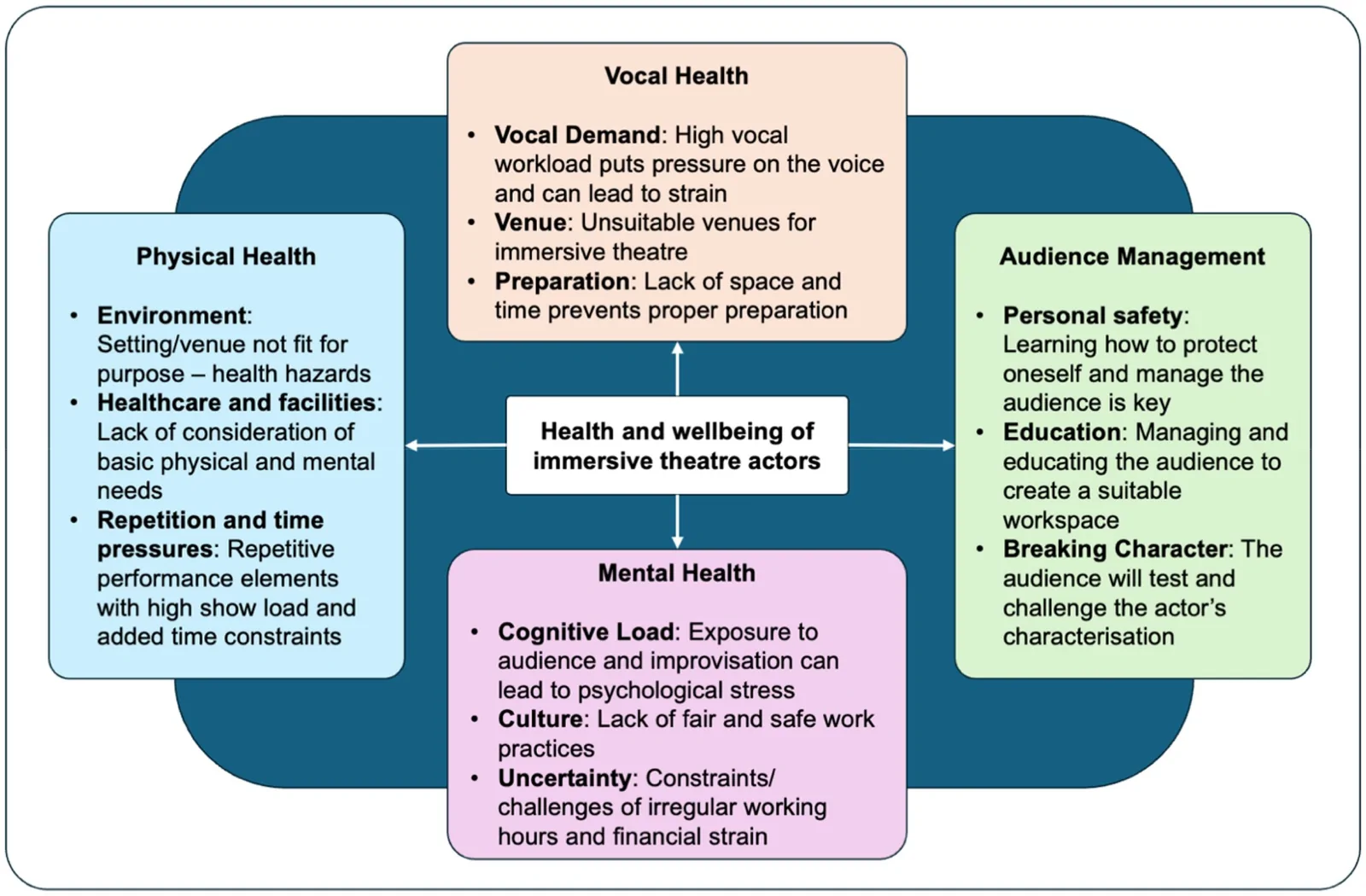
Thematic map illustrating the main themes, sub-themes and codes.

**Table 1:** Participant information

Code	Age	Gender	Ethnicity	Experience (years)
P01	27	F	White	5
P02	55	M	White	25
P03	27	F	White	5
P04	50	F	White	1
P05	36	M	White	4
P06	46	M	White	2
P07	30	M	White	7
P08	34	M	White	7
P09	44	M	White	8
P10	42	F	White	10

**Table 2:** Themes, sub-themes and quotations

Subtheme	Quotations
**Vocal health**	
Vocal demand	‘It’s very, very hard on your vocal health when you’re trying to do four shows a day, four days in a week’ [P09] ‘The show was fifty minutes long, but it was a one actor experience. So, it’s basically fifty minutes of dialogue’ [P07]
Venue	‘These spaces are not made with acoustics in mind’ [P03] ‘It was dusty, it was dirty… the inherent dust was always there’ [P04]
Preparation	‘We’re very rarely given enough time before our shift starts to do a proper vocal and physical warm up’ [P03] ‘When you arrive at a venue, it’s often already up and running. So, if you’re in the next show, it’s not like a play where there’s a place where you can go to warm up’ [P05]
**Mental health**	
Cognitive load	‘You’ve got to make sure that you’re engaging everyone constantly and ready to respond to anything that’s being shouted at you’ [P01] ‘With big groups, there is a huge level of anxiety because you don’t know if anyone in that group is going to misbehave or challenge you. It’s exhausting. Like it is mentally exhausting’ [P10]
Culture	‘We would definitely be made to feel like we were the problem’ [P03] ‘A lot of the immersive is run by businesses who are about money, and they see actors mostly as a means to an end, warm props who are expendable and interchangeable’ [P10]
Uncertainty	‘I didn’t see a contract when I first joined. It’s just freelance, like zero hours. I don’t even know what my contract actually is like’ [P08] ‘They’re literally doing their tickets on a week-to-week basis. In some cases, they’re cancelling shows the week before’ [P01]
**Physical health**	
Environment	‘It’s [the set] not meant to handle dozens upon dozens of people in multiple shows a night, multiple times a week for years. So, things break all the time’ [P08] ‘It’s this whole basement of dark flickering lights and stuff hanging out the ceilings. You can’t tell where grill mesh walls are…and I did have an accident’ [P06]
Healthcare & facilities	‘I don’t know if we even have an ice pack’ [P08] ‘Dressing rooms were overcrowded. There were two dressing rooms for all fifty performers on a rotational basis’ [P04]
Repetition and time pressures	‘There’s the turnaround, you’ve got like a minute and a half. We had to get back to the start so, you got to run’ [P04] ‘The turnaround on the shows is what I struggle with the most. It’s back-to-back with the tracks… you come off one and you go straight into the next’ [P01]
**Audience management**	
Personal safety	‘It’s not like theatre, the audience, aren’t safely in the seats, they’re all around you. So, if someone doesn’t like or is negative to what you’re doing, you know about it immediately’ [P02] ‘You’ve got wild, great apes set loose in an environment which is already breaking social norms’ [P09] ‘I was like cornered by a group of men…. that’s when it can get really frightening’ [P03] ‘I’ve had audiences like properly square up to me to engage in fights and I’ve seen them actually get into fights with other people as well. So, it’s really intense’ [P07]
Education	‘There’s just a lot of ignorance and inexperience on the audience part, which is…a lot of burden on us to teach … what’s, OK what’s not OK’ [P08] ‘People are still sort of learning what it [immersive] is’ [P01]
Breaking character	‘People see it as a challenge to break the actors. I’m gonna go in and I’m gonna break the actor’ [P01] ‘They’re not saying it to you personally. They’re trying to make you break character. So, they’re trying to make you vulnerable’ [P02]

The role requirements in immersive theatre can be highly voice-intensive, often resulting in vocal overload. Additional environmental factors further exacerbate pressures and may increase the risk of voice fatigue and other vocal symptoms.

Vocal demand (Subtheme 1) refers to the vocal requirements of the actor in a given scenario over a specified period and is influenced by the environment, content, audience profile, and social/emotional context [15]. Participants described a high vocal demand with multiple shows per day; infrequent breaks; repetitive dialogue; sustained projection over background noise; and accent work or extreme voice techniques such as screaming or shouting. Venues (Subtheme 2) were frequently repurposed industrial spaces not originally designed for performance, such as warehouses or basements with poor acoustics, air quality and temperature regulation. Actors were exposed to environmental irritants, including dust, chemical haze or ongoing building works. Wide variation in acoustics across spaces within a single venue added to vocal strain. Preparation (Subtheme 3) was consistently inadequate prior to the show opening, often with a lack of full on-site technical rehearsals. Short call times (specified time when actors are required to be present on set) prevented proper vocal warm-ups, and the lack of understudies and sick pay fostered presenteeism.

The removal of the fourth wall changes the psychological experience of performance. Actors in immersive theatre are directly exposed to the audience, with no defined boundary or psychological shelter. This, combined with a lack of fair work practices and irregular hours, negatively impacts mental health.

Cognitive load (Subtheme 4) was a central concern. Actors must simultaneously deliver key scripted elements alongside improvised material in response to unique audience interactions. They must manage unpredictable events, respond to security issues and support the audience in navigating the space safely. Emotionally demanding roles can lead to ‘boundary blurring’, where an actor struggles to separate their fictional characters from their real self. Immersive theatre worlds can be highly stimulating, featuring complicated sets, dynamic props, noise and lighting effects. This may be overstimulating and neurobiologically exhausting, leading to stress, burnout or symptoms of occupational trauma exposure. Workplace culture (Subtheme 5) compounded the burden. Company managers play a key role in supporting the health and wellbeing of actors. They are responsible for training, scheduling, cover arrangements and safeguarding. However, participants widely reported that raising concerns often led to actors being dismissed, ignored or excluded. A lack of clear safeguarding policies and procedures creates barriers for the actor needing to communicate their mental health status or seek help. Fear of being ‘blacklisted’ or labelled difficult enforced a culture of silence around health issues. Uncertainty (Subtheme 6) about pay and contracts added further pressure. Most actors work zero-hour contracts and are paid a ‘show fee’ or a day rate. Pay is low, companies are often slow to forward payment and rotas could be unpredictable and change significantly at short notice. This led to financial concerns and an inability to rely on a regular income.

Participants believed immersive theatre roles carried a higher physical injury risk than working in conventional theatre settings due to a variety of environmental factors and a lack of healthcare and basic facilities.

The environment (Subtheme 7) was frequently described as hazardous. Sets and props were not designed to withstand multiple shows and repeated audience traffic, creating trip hazards and increased injury risk. Actors may have additional manual handling tasks due to fewer stage crew members employed. The theatre environment presents an elevated risk of head injury [16], and the audience moving unpredictably through the performance space increases the risk. Combat and dance scenes were harder to execute safely for the same reason, leading to collisions. This was compounded if audience members were inebriated or if children were inadequately supervised. Exposure to loud music or other noise increases the risk of permanent hearing damage. There were concerns about the duration and intensity of sound exposure, with no provision for ear protection. Healthcare and facilities (Subtheme 8) were minimal or absent. While first aiders were present during performances, unlike traditional theatre, there was no other healthcare provision, such as physiotherapy clinics, health screening or monitoring. A lack of basic welfare facilities to dress, wash and find refreshments causes stress, fatigue and encroaches on break times if actors must travel significant distances to find resources. Repetition and time pressures (Subtheme 9) add further risk, where actors worked within tightly timed show formats. This led to time-pressured behaviour, higher levels of stress, a narrow margin for errors and inadequate recovery time. Producers or directors lacking performance industry experience were felt to hold unrealistic expectations about safe working hours, rehearsal time and break patterns.

Immersive theatre actors work in close proximity to the audience in a shared performance space. This defining feature can be a significant occupational hazard, and actors described self-protection strategies and audience management approaches to help create a safer workspace.

Personal safety (Subtheme 10) emerged as a serious and under-recognized concern. Audience reactions to the immersive theatre space vary considerably depending on a range of personal factors. Those used to consuming theatre passively may struggle to detach fiction from reality. Participants described incidents where audience members verbally, physically or sexually harassed actors or other audience members. If an actor portrayed a character displaying offensive or outdated attitudes it could attract aggressive responses from the audience. Female actors felt particularly vulnerable and adapted their voice, delivery and physical positioning to avoid confrontation. Poor audience management practices fostered problematic audience behaviour. This included: inadequate audience vetting and induction; ineffective marketing creating unrealistic expectations; high volume and rate of audience admission; and the promotion of excessive alcohol consumption. When safeguarding procedures were clear, and security staff responded swiftly, actors felt safer. When they were absent or slow, it led to increased stress and anxiety for the actors. In most cases, actors were not offered a debrief or support following critical incidents. from the audience

Education (Subtheme 11) depleted the cognitive and emotional resources of the actors, who were burdened with teaching the audience immersive world etiquette or game rules. Breaking character (Subtheme 12), where an actor accidentally or purposefully steps away from their character’s persona and interacts with the audience as their ‘real self’, was discussed by participants. Audience members may deliberately attempt to destabilize the actor, whether out of curiosity, searching for connection, being playful or a desire for control. Most actors were skilled at handling this; however, for inexperienced actors, it may be a source of stress or feel like a violation of professional integrity.

## Discussion

This is the first study to examine the occupational health impact of working in immersive theatre performance on actors. Four principal concerns of vocal overload, physical injury risk, mental health issues and audience-related pressures were identified.

This qualitative study using semi-structured interviews captured detailed, participant-led descriptions of the immersive theatre working environment. However, the sample was small and self-selected, so it may not represent the full range of experiences across the sector. Self-selection may have biased the sample towards those with negative experiences. The single method of data collection may have excluded alternative perspectives, and member checking was not undertaken, which may have led to misrepresentation of participant experiences. In addition, all participants were classified as White, which limits the transferability of findings and highlights the need for more diverse sampling in future research. Quantitative studies with larger samples are needed to establish the prevalence and severity of health problems, and longitudinal research would help establish the impact on health over time. The effectiveness of targeted interventions such as structured warm-up protocols, mandatory debriefs and audience management training requires prospective evaluation. In addition, studies exploring the perspectives of company managers and producers would gain insight into organizational barriers to supporting actor health.

The life of an actor has been described as precarious, with high risks and often minimal rewards despite significant investment in their vocation [17]. Precarious employment is associated with poor mental health [18], so, predictably, there is established evidence of elevated depression and psychological distress in actors [1,2]. A lack of psychological safety is a broad issue across theatre. A recent survey found that only 4% of theatre actors felt safe reporting concerns due to fear of negative consequences [19]. The adverse work culture and financial uncertainty reported by immersive theatre actors reflect this wider pattern, with heightened cognitive load compounding these pressures. The performing arts and entertainment trade union Equity has identified the immersive sector as a key campaign priority for fair pay, setting up a member network and signing term agreements with leading immersive companies [20]. Managers with relevant performance industry experience should foster a supportive organizational culture that enables actors to raise health concerns without fear of professional reprisal. To manage boundary blurring and role-related emotional distress, awareness training, mandatory debriefing after critical incidents, access to specialized mental health support and training in psychological self-regulation can support wellbeing [1].

Actors are vocal athletes and must accommodate intense vocal loads. Subtle alterations in vocal function can have a devastating impact, leading to cancelled performances [21]. High vocal demand is associated with an increased risk of voice disorders [22,23], and participants in this study highlighted excessive demand compared to other genres. Effective scheduling, understudy cover and contingency planning would reduce the risk of vocal overload and presenteeism. Airborne irritants such as dust, smoke and glycol or mineral-based special effects are known risk factors for voice and respiratory problems in actors and should be eliminated [2,24]. If the venue’s dimensions and acoustics prevent the actor from safely projecting their voice, amplification or adjustments to the space are required. The efficacy of vocal warm-up for preserving voice is well established [25], so call times should allow for adequate preparation. Voice use for dramatic intensity is comparable to extended periods of shouting [26]. Vocal training has a protective effect for actors, particularly when intense vocal practices are required for a role [27]. Rehearsing with a suitably qualified vocal coach allows actors to manage these manoeuvres safely.

A systematic review reported that musculoskeletal injuries were common among actors, with an average of 1–2 injuries per year [2]. Compared with normative data, actors also show a higher prevalence of neck, shoulder, back and upper-limb pain [23], and participants in this study described additional risks unique to immersive theatre related to the venue, show format and healthcare support. To mitigate this, a comprehensive health and safety risk assessment should examine audience capacity, ventilation, sound exposure, flooring, exits, staircases, sets, props, costumes, footwear, welfare facilities, scene changes and manual handling [4,28,29]. In addition, stage weapons and combat scenes should be properly risk-assessed and choreographed in accordance with HSE Guidelines on the management of firearms and other weapons in productions [4,30]. Head injuries need to be reported, documented and have concussion screening and care procedures implemented. Since 2008, the music and entertainment sector has been legally required to comply with The Control of Noise at Work Regulations 2005. Actors exposed to sound hazards require personal ear protection or other sound-reduction measures to reduce the risk of harm [4,31]. On-site physiotherapy clinics decrease work-related musculoskeletal disorders by providing health screening, monitoring and treatment interventions [32].

Immersive theatre actors may be exposed to challenging audience reactions such as anger or aggression [29]. The simultaneous requirement to create authentic connections with the audience whilst remaining constantly alert to potential threats from the same people is psychologically complex and can cause elevated stress and hypervigilance. Effective audience management procedures reduce errant audience behaviour and provide a sense of safety for the actors. Audience autonomy needs to be balanced with clear boundary-setting and embedding effective consent mechanisms in the immersive space [10]. Realistic marketing and proactive onboarding guidelines related to accessibility, safety and behaviour should be included. A rehearsal phase with a test audience helps identify problems, and actor training involving audience interaction role-play helps prepare actors for difficult scenarios. Admission throughput should be balanced, audience members displaying aggressive or inebriated behaviour should be removed swiftly by trained security personnel, and critical incidents should be reported [29]. The Creative Industries Independent Standards Authority (CIISA) has developed a framework for professional standards, relating to safe and inclusive working environments, open and accountable reporting mechanisms and a responsive learning culture [33]. This framework highlights the need for clear reporting procedures for actors experiencing physical, sexual or verbal harassment. Government legislation requires employers to take reasonable steps to prevent harassment at work, including sexual harassment, bullying and other harmful behaviour. Policies developed in consultation with a recognized trade union, such as Equity, would help to identify and manage risk.

Existing occupational health frameworks do not sufficiently address the genre-specific demands of immersive theatre. Robust policies and procedures that comply with government guidance and align with creative industry standards should be implemented to support the actors’ health and create a safer working environment.

## Supplementary Material

kqag086_Supplementary_Data
