# Supplementary material for: The impact of working in immersive theatre on actors’ health
Source: Occup Med (Lond). 2026 Sep 4;76(7):479–85. doi: 10.1093/occmed/kqag086 (PMC13740512; doi:10.1093/occmed/kqag086)
Supplement: Supplementary file 1 [file kqag086_supplementary_data.doc]

**APPENDIX I**

***Appendix I: Table showing interview schedule***

| 1 | Could you describe the training and career path that led to you working in immersive theatre? |
| --- | --- |
| 2 | What do you enjoy about immersive theatre roles? |
| 3 | How do these roles differ from more traditional theatre or other performance roles? |
| 4 | Have you suffered a musculoskeletal injury whilst working in immersive theatre? |
| 5 | Does working in immersive theatre roles impact your vocal health? |
| 6 | Has your mental health been affected by working in immersive theatre? |
| 7 | Are there aspects of the working environment unique to immersive theatre that can impact health and wellbeing |
| 8 | Describe your experience of interacting and being near the audience? |
| 9 | Could you describe any policies or procedures that aim to support your health, wellbeing and safety whilst working in immersive theatre? |
| 10 | What healthcare support do you receive whilst working in immersive theatre? |
